# Digital Health Technologies Applied by the Pharmaceutical Industry to Improve Access to Noncommunicable Disease Care in Low- and Middle-Income Countries

**DOI:** 10.9745/GHSP-D-22-00072

**Published:** 2022-10-31

**Authors:** Anne Christine Stender Heerdegen, Carlotta Maria Cellini, Veronika J. Wirtz, Peter C. Rockers

**Affiliations:** a Department of Global Health, Boston University School of Public Health, Boston, MA, USA.

## Abstract

**Background:**

There is limited research on how digital health technologies (DHTs) are used to promote access to care for patients with noncommunicable diseases (NCDs), particularly in low- and middle-income countries (LMICs). We describe the use of DHTs in pharmaceutical industry–led access programs aimed at improving access to NCD care in LMICs.

**Methods:**

The Access Observatory is the largest publicly available repository containing detailed information about pharmaceutical industry—led access programs targeting NCDs. The repository includes 101 access program reports submitted by 19 pharmaceutical companies. From each report, we extracted data relating to geographic location, disease area, beneficiary population, use of DHTs, partnerships, strategies, and activities. Data were analyzed descriptively using SAS Statistical Software and categorized according to the World Health Organization Digital Health Classification Framework.

**Results:**

A total of 43 access programs (42.6%) included DHTs. The majority of programs using DHTs were clustered across sub-Saharan Africa (72.1%) and targeted cancer (60.5%) followed by metabolic disorders (39.5%). The applied DHTs mostly related to program strategies on health service strengthening (74.4%) and community awareness (41.9%) and were largely directed toward health providers, followed by data services and clients. Only a few DHTs were used for health system management. To promote access, most DHTs focused on improving data collection, management, and use (51.1%); building health provider capacity through training (37.2%); and providing targeted patient information (34.8%).

**Conclusion:**

The range of DHTs applied by the pharmaceutical industry offers opportunities for more effective access to NCD care. Transparent reporting on DHT use and its contributions to access programs’ achievements may reduce duplicative and redundant efforts and provide learnings for private and public stakeholders that may contribute to greater access to NCD care in LMICs.

## Abstract (precis)

Digital health technologies applied by the pharmaceutical industry offer opportunities to improve access to care for patients with noncommunicable diseases in low- and middle-income countries.

## Teaser

**Key Findings:**

- Pharmaceutical industry–led access programs have benefited from the application of digital health technologies (DHTs) to educate and empower patients with noncommunicable diseases (NCDs) to make better-informed decisions about their health and to enhance service delivery through telemedicine, telepathology, and capacity building of health care workers.
- DHTs applied by global pharmaceutical companies in their access programs that aim to improve NCD care in low- and middle-income countries are primarily implemented across sub-Saharan Africa and mostly directed toward health providers. Digital technologies targeting data services and patient populations were also commonly applied to respectively collect, manage, and use data, as well as to enhance patients’ disease management, awareness, and medication adherence.

**Key Implication:**

- Evidence on pharmaceutical industry–led DHTs may enable national stakeholders to identify opportunities to promote access to NCD care in various health system contexts and prevent siloed work across sectors. Moreover, it may provide guidance and orientation to policy makers in identifying duplicative and redundant digital health efforts that may overburden local health systems.

## INTRODUCTION

In many low- and middle-income countries (LMICs), there is an increasing burden of noncommunicable diseases (NCDs), such as cardiovascular disease, cancer, and diabetes. However, few health systems in LMICs are organized to meet the needs of NCD care, which includes long-term and continuous care.^1^ People with limited resources from poor households in remote areas are particularly vulnerable in terms of accessing NCD care due to treatment costs and indirect costs related to obtaining care due to limited health services availability, such as shortages of health facilities, properly trained and equipped health care professionals, medicines, and equipment.^1^^,^^2^

Reducing the burden of NCDs and achieving universal health coverage are among the United Nations’ 2030 Sustainable Development Goals, and the pharmaceutical industry plays an important role in achieving these goals. As a result of the global agenda combined with greater attention from investors, employees, and patients, the pharmaceutical industry has expanded its efforts to strengthen its environmental, social, and corporate governance initiatives.^3^^,^^4^ Among other goals, the social initiatives focus on enhancing access to medicines and care in LMICs.^5^ The current crisis of global vaccine inequity brought on by the coronavirus disease (COVID-19) pandemic has increased the public’s focus on such initiatives.^6^

Several access initiatives focusing on NCDs in LMICs have been established by pharmaceutical companies in recent years.^5^ These include Novartis Access, which provides a portfolio of medicines for NCDs at reduced prices in multiple lower-income countries; Bristol Meyer Squibb’s Project ECHO for Cancer Care, which increases workforce capacity to provide best-practice specialty care and reduce health disparities in South Africa; and Novo Nordisk’s Changing Diabetes in Children, which takes on a systemic approach to address access barriers to diabetes care in a range of LMICs. These programs, as well as many more pharmaceutical industry–led access programs, have benefited from the application of digital health technologies (DHTs) to educate and empower patients to make better-informed decisions about their health and to enhance service delivery through telemedicine, telepathology, and capacity building of health care workers. In this article, we refer to pharmaceutical-led access programs as pharmaceutical companies’ efforts to improve access to NCD medicines and care in LMICs by addressing a wide range of health system access barriers.^4^

Pharmaceutical industry–led access programs have benefited from the application of DHTs to inform, educate, and empower patients to make better-informed decisions about their health.

The World Health Organization (WHO) and others in the global health community have recognized DHTs as having a large potential to promote access to health care in LMICs.^7^^–^^9^ Digital health solutions typically leverage the Internet, social media platforms, and mobile phone usage. In 2019, more than 4 billion people used the Internet. More than 7 billion people lived in an area that is covered by a mobile-cellular network, and more than 6 billion smartphones are in use around the world.^10^ Nonetheless, shortcomings prevail in research on the application of DHTs in the health sector and particularly how these can work to reduce the NCD burden in LMICs.^7^^,^^11^ The lack of research limits potential opportunities to learn about effective DHTs targeting NCDs and may hinder effective governance of these, which is critical in a time of continuous growth in DHTs.^12^

In this article, we aim to describe the use of DHTs in pharmaceutical industry–led access initiatives targeting NCDs in LMICs. The pharmaceutical industry plays an important role in digital health solutions targeted at NCDs with its plentiful resources and business agenda to enhance access in emerging markets. Our findings may shed light on potential pioneer technologies targeting NCDs that may be beneficial for national stakeholders in terms of strengthening health systems and reaching the health-related Sustainable Development Goals. Moreover, these findings may provide guidance and orientation to policy makers in terms of identifying duplicative and redundant digital health efforts that may overburden local health systems.

## METHODS

### Definition of DHT

There are many definitions of digital health. According to WHO, the term digital health is rooted in eHealth, which is defined as “the use of information and communication technology (ICT) to improve health.”^7^ Digital health refers to a broad range of technologies that can make health information, health services delivery, and diagnostics more accessible for hard-to-reach populations.^11^ The technologies include telehealth, mHealth, wearable devices, self-monitoring medical devices, digital diagnostics, digital therapeutics, and clinical decision support solutions, as well as the advanced algorithms and artificial intelligence systems that support these.

### The WHO Digital Health Classification Framework

We use the WHO Digital Health Classification Framework to describe DHTs. The framework aims at providing a shared language to articulate functionalities of digital technologies for health. It includes 4 main categories of interventions for: (1) clients, (2) health care providers, (3) health system or resource managers, and (4) data services.^13^ Each category includes subcategories that further specify the function of the health technology (Supplement 1). Clients are members of the public who are potential or current users of health services. Health care providers are members of the health workforce who deliver health services. Health system or resources management involves the administration and oversight of public health systems. Interventions target managerial functions related to supply chain management, health financing, and human resource management. Data services relate to data collection, management, use, and exchange. A DHT can be classified within multiple WHO categories.

### The Access Observatory Taxonomy of Program Strategies

In addition to using the WHO Framework to describe DHTs, we describe the functionality of the applied DHTs by using Rockers et al.’s taxonomy of program strategies, which includes 11 strategies that are organized into 4 broad categories (Box).^4^ Access programs can address access barriers by using a variety of health system approaches, including community, system, production, and pricing strategies.

BOX **Taxonomy of Program Strategies**

**Community strategies**

Community awareness and linkage to care: Programs providing communities and patients with health-related information on disease prevention and treatment or improve links between patients and the health care system.

**System strategies**

Health service strengthening: Programs designed to improve the availability, affordability, and quality of health services.

Health service delivery: Programs designed to deliver health services, such as screening, diagnosis, and treatment, directly to the patient.

Supply chain: Programs designed to improve medicine supply chains, to improve availability and lower costs.

Financing: Programs designed to improve health financing systems and reduce catastrophic health expenditure.

Regulation and legislation: Programs designed to improve government coverage and access to treatments and/or improve in-country regulatory processes through, for example, advocacy, training, and infrastructure.

**Production strategies**

Manufacturing: Programs designed to build capacity for medicine production.

Product development and research: Programs designed to support product (i.e., medicines or devices) development and research.

Licensing agreements: Programs designed to facilitate the manufacture, importation, sale, or use of medicines through legally binding relationships, including voluntary licensing agreements and technology sharing.

**Price strategies**

Price scheme: Programs designed to increase the affordability of medicines for individuals and health care systems through a change in the price via subsidies or other means (excluding donations).

Medicine donation: Programs designed to increase the availability and/or affordability of medicines through direct donation of medicines and other health care products to countries, health institutions, or nongovernmental organizations.

Adapted from Rockers et al.^4^

### Data Source

The Access Accelerated is an initiative established by 23 biopharmaceutical companies, the World Bank, and the Union of International Cancer Control. Together, they are committed to expanding their efforts to improve access to NCD prevention, care, and treatment in LMICs.^14^ The Access Observatory, an integral part of Access Accelerated, is the largest publicly available repository of detailed information about pharmaceutical industry–led access programs.^4^ The repository, which is the primary data source of this study, contains information on access programs that have been submitted by the respective pharmaceutical companies between 2017 when it was established and 2021 when this study was conducted. The repository contains detailed program descriptions and information on the countries of program implementation, disease focus, beneficiary populations, involved stakeholders and partners, access program strategies, and activities including the use of DHTs, program indicators, and measurements of program inputs, outputs, and outcomes.

### Data Extraction

Each program report registered within the repository was reviewed independently by the first (ACSH) and second author (CMC) of this publication. A spreadsheet was used to extract information systematically from the repository program reports on the following variables: whether the program applied DHTs to improve access to prevention, care, and treatment of NCDs; a description of the DHT, including countries of implementation and targeted disease area; stakeholders and partnerships involved in implementing the DHT; which program strategy the DHT related to according to Rockers et al.’s program strategy taxonomy (Box)^4^; and classification of the DHT according to the WHO Digital Health Classification Framework (Supplement 1).^13^ For programs employing DHTs, the access strategy relevant to the DHT was counted. If the DHT was applied in multiple access strategies, they were all counted. WHO classifications were counted similarly.

Discrepancies in the 2 independently completed spreadsheets were discussed between the first and second author until agreement was reached. In cases of doubt, company and program websites were reviewed to learn more about the program and application of the specific DHT.

### Analysis

The final spreadsheet with content agreed to by the first and second authors was imported into the statistical software SAS Studio 3.81. Subsequently, descriptive frequency analyses were performed to generate an overview of the range of technologies, their countries of implementation, targeted disease areas, applied strategy, and WHO classification. Bivariate analyses were performed to assess differences between access programs employing DHTs and programs where DHTs are not applied. Differences in proportions between groups by DHT use were tested with a Fisher’s exact test, which is recommended for small-sized samples.^15^

## RESULTS

A total of 19 companies have registered their access programs in the repository. The majority of companies reported on multiple access programs resulting in 101 program reports for review. The registered programs were initiated between 2009 and 2020, and 61 remain active at current date.

Of the 101 programs, 43 programs (42.6%) from 12 companies (63%) included a DHT as part of their access program. An overview including descriptions of each of the DHTs, relevant strategies, and WHO classifications can be found in Supplement 2.

The Table shows that 31 programs (72.1%) using DHTs took place in sub-Saharan Africa, with most in Kenya and South Africa, and 14 programs (32.6%) using DHTs were implemented in South Asia, mostly in India (Supplement 2). Nine programs (20.1%) using DHTs were implemented across multiple regions, including 3 programs implemented in high-income countries. There were no statistically significant differences in countries of implementation between access programs employing DHTs versus programs without DHTs.

**TABLE.** Characteristics of Digital Health Technologies Used in Pharmaceutical Industry–Led Access Programs

	**Programs With a DHT, No. (%)** **(n=43, 42.6%)**	**Programs Without a DHT, No. (%)** **(n=58, 57.4%)**	***P* Value**
Region of implementation			
Sub-Saharan Africa	31 (72.1)	34 (58.6)	.21
South Asia	14 (32.6)	23 (39.7)	.53
Latin America and Caribbean	7 (14)	12 (20.7)	.61
Middle East and North Africa	5 (11.6)	8 (13.8)	1.0
East Asia and Pacific	4 (9.3)	4 (6.9)	.49
Europe and Central Asia	3 (7.0)	7 (12.1)	.51
Targeted disease area			
Cancer	26 (60.5)^b^	44 (75.9)^b^	-
Breast	4 (9.3)	10 (17.2)	.38
Lung cancer	5 (11.6)	1 (1.7)	.08
Other	17 (39.5)	33 (56.9)	.16
Metabolic disorders	17 (39.5)^b^	5 (8.1)^b^	-
Type 1 diabetes	5 (11.6)	1 (1.7)	.08
Type 2 diabetes	12 (27.9)	3 (5.2)	.03^b^
Cardiovascular diseases	11 (25.6)^b^	8 (13.8)^b^	-
Cardiovascular, general	3 (7.0)	3 (5.2)	.70
Hypertension	7 (16.3)	5 (8.6)	.35
Asthma	1 (2.3)	0	.43
Mental and neurological disorders	5 (11.6)^b^	7 (12.1)^b^	-
Mental health, general	4 (9.3)	7 (12.1)	.76
Alzheimer’s	1 (2.3)	0	.43
Mother and child health	3 (6.8)^b^	0^b^	.07
Respiratory disease	0^b^	2 (3.4)^b^	.51
Musculoskeletal diseases	1 (2.3)^b^	1 (1.7)^b^	-
Osteoporosis	1 (2.3)	0	.43
Arthritis	0	1 (1.7)	1.0
Other NCDs	4 (9.3)^b^	13 (22.4)^b^	.11
Access strategy^a^			
Health service strengthening	32 (74.4)	44 (75.9)	1.0
Community awareness and linkage to care	18 (41.9)	39 (67.2)	.03^b^
Health service delivery	11 (25.6)	29 (50)	.02^b^
Supply chain	3 (7.0)	3 (5.2)	.69
Product development and research	1 (2.3)	0	.43
Financing	0	4 (6.9)	.13
Price scheme	0	10 (17.2)	<.01^b^
Regulation and legislation	0	3 (5.2)	.26
Medicine donation	0	9 (15.5)	<.01^b^
WHO Digital Health Classification^c^			
Health care providers	35 (81.4)	-	
Data services	24 (55.8)	-	
Clients	18 (41.8)	-	
Health system management	9 (20.9)	-	

Abbreviations: DHT, digital health technology; NCD, noncommunicable disease; WHO, World Health Organization.

^a^Each program and DHT can apply to multiple strategies.

^b^Statistically significant (2-sided *P*<.05). Differences between groups were tested with a Fisher’s exact test.

^c^Each DHT can apply to multiple classifications.

The disease areas targeted by the DHTs were primarily cancer (26, 60.5%) or metabolic disorders (17, 39.5%), including type I and type II diabetes. Industry-led programs targeting metabolic disorders, particularly type 2 diabetes, were significantly more likely to apply DHTs than not (*P*=.03).

Of the 11 different program strategies listed in the Access Observatory taxonomy (Box), 5 strategies were targeted by programs applying DHTs (Table). The majority of these used DHTs as part of their strategies focused on health service strengthening (32, 74.4%) or community awareness and linkage to care (18, 41.9%). Nonetheless, there appeared to be significantly more programs without DHTs targeting community awareness and linkage to care (*P*=.03), health service delivery (*P*=.02), price scheme (*P*<.01), and medicine donation (*P*<.01).

Digital health solutions applied in the programs mostly targeted health care providers (35, 81.4%), followed by data services (24, 55.8%), clients (18, 41.8%), and health system management (9, 20.9%).

**FIGURE 1:**
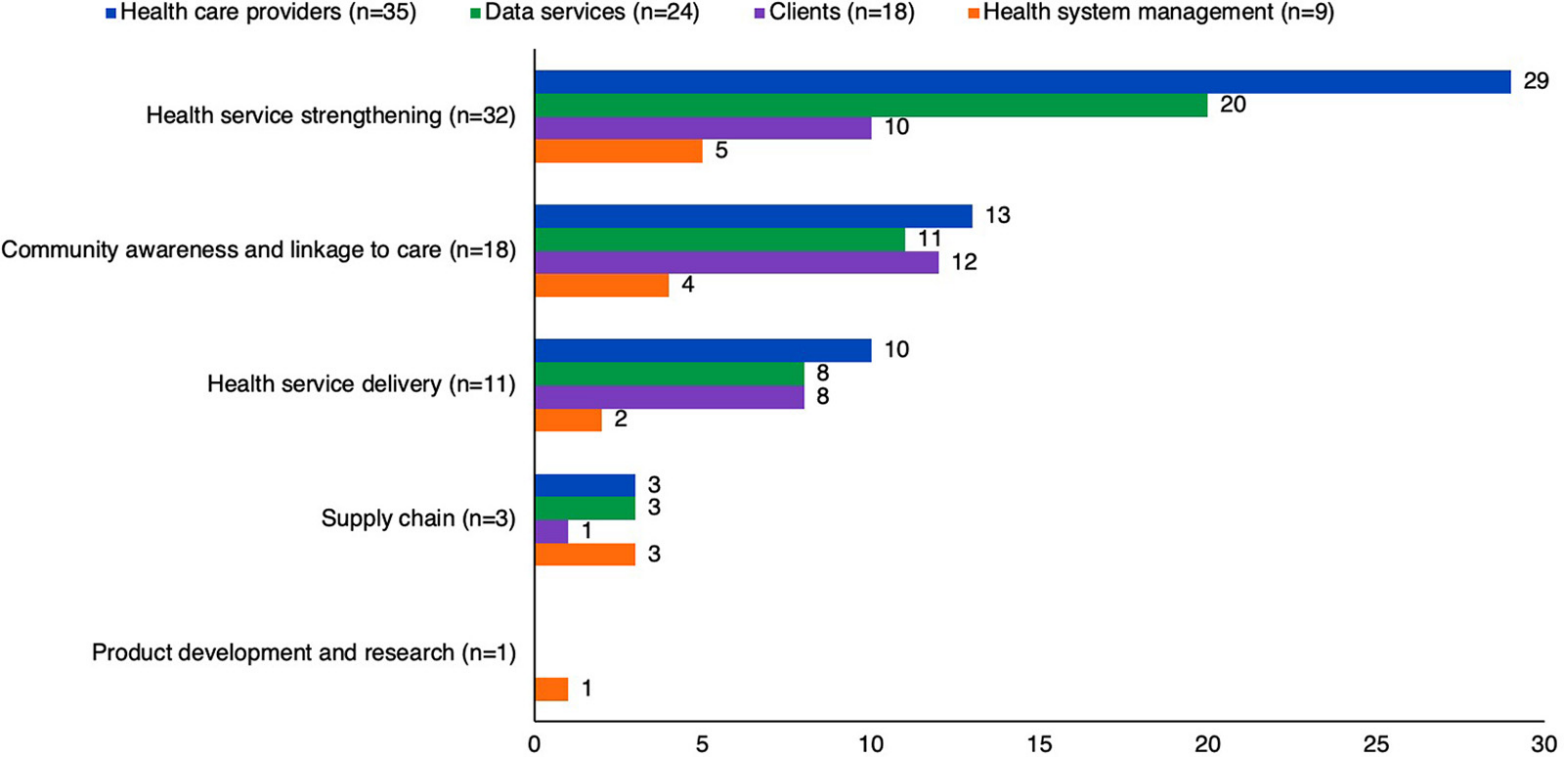
World Health Organization Digital Health Classifications by Program Strategy

Figure 1 merges the Access Observatory and WHO Digital Health Classification taxonomies. It demonstrates that access programs aiming to enhance access to NCD care through health service strengthening, community awareness, and health service delivery mostly applied DHTs directed toward health care providers. Data service–focused DHTs were also applied relatively often for health services strengthening (n=20), and client-focused DHTs (n=12) were used for community awareness and linkage to care.

Programs aiming to enhance access to NCD care through health service strengthening, community awareness, and health service delivery mostly applied DHTs directed toward health care providers.

### DHTs Targeting Health Providers

**FIGURE 2:**
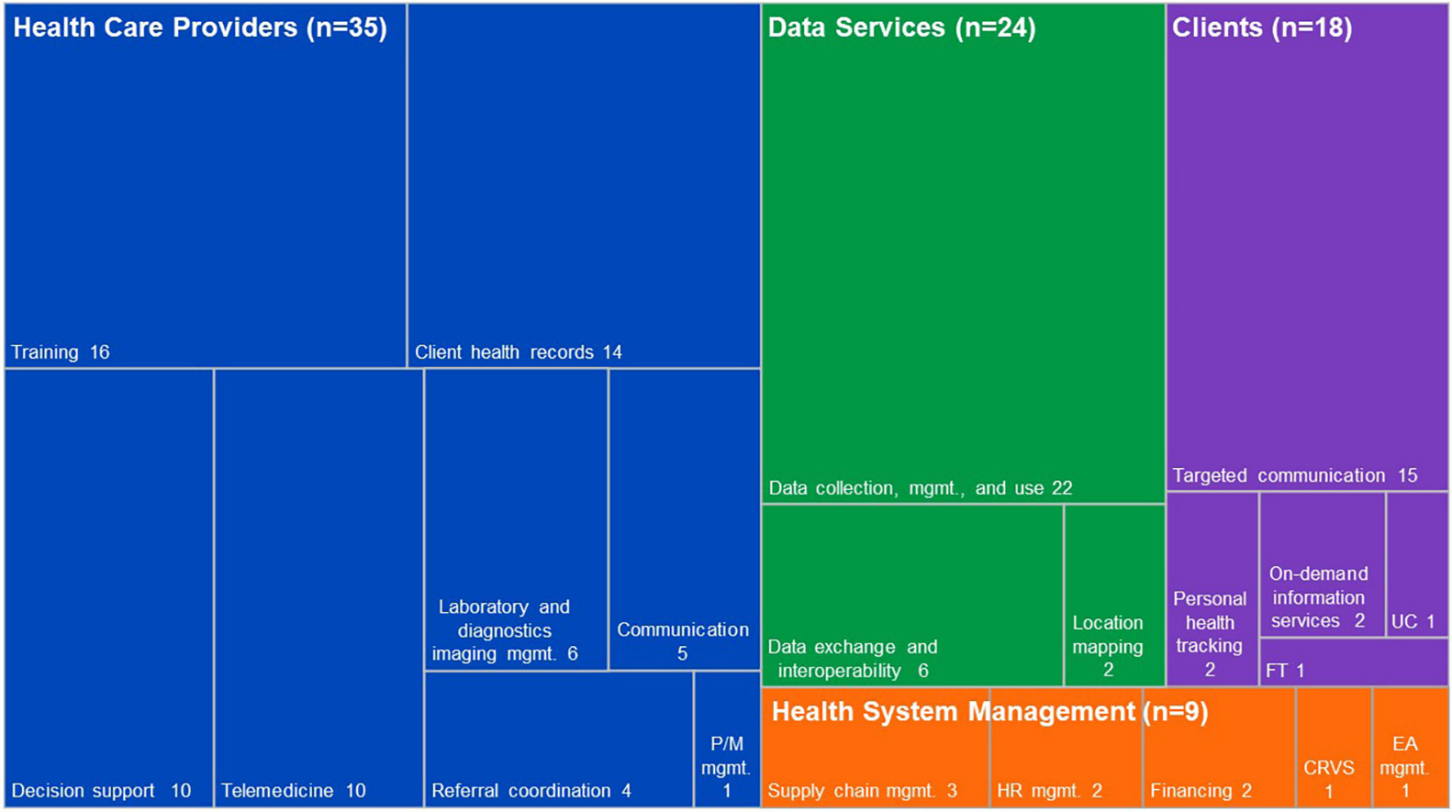
Treemap of Digital Health Technologies According to the World Health Organization Digital Health Classification Framework^a^ Abbreviations: CRVS, civil registration and vital statistics; EA, equipment and asset; FT, financial transactions; HR, human resources; mgmt, management; P/M, prescription/medication; UC, untargeted communication. ^a^The different colors represent the 4 World Health Organization categories and their subcategories. The size of the rectangle represents a quantitative value, namely the number of programs targeting the subcategories, and is proportional to the quantitative value.

Figure 2 shows that the majority of DHTs targeting health care providers involved health provider training (n=16). One DHT used for health provider training by multiple access programs across Kenya was LEAP, an interactive mHealth platform that delivered chronic NCD (diabetes, cardiovascular diseases, and cancer) care eLearning content through text messages and audio files using simple phone technology, as well as animations and illustrations to smartphones (Supplement 2). Other examples of digital training include virtual workshops to upskill primary health care providers on the topic of mental and neurological disorders (e.g., Sanofi Mental Health Program FAST-South Africa) and eLearning platforms with online modules that health care providers can complete at their own pace with interactive webinars organized by local psychiatrists (e.g., Sanofi FAST eLearning).

In addition, client health records were the target of DHTs in multiple access programs (n=14) aiming at health service strengthening and health service delivery. These DHTs involve electronic patient medical record systems that securely document patient data that enable health providers to provide accurate referrals and follow-ups and to facilitate patient management and treatment, particularly in areas of oncology, diabetes, and cardiovascular diseases. As an example, 2 of these programs in Kenya used the Mobile Jamii Afya Link to enable community workers to accurately and efficiently collect and manage NCD patient data at the household, community, and medical facility levels. Electronic systems for health records were among other methods used to improve tracking of disease incidence and burden of disease cases at various hospitals, as well as to inform policy making.

DHTs enabling health care provider decision support were also applied frequently (n=10). These include electronic risk factor screening tools for lung cancer and mental health; electronic medical records with decision-support functions that provide relevant prompts and alerts (e.g., for follow-up to decrease time along the patient journey), and electronic classification tools that assess and alert health providers about patients’ risk of cancer treatment abandonment. Other decision support DHTs include digital protocols for treatments, patient information, and platforms to seek advice from specialists, such as psychiatrists and oncologists.

DHTs enabling health care provider decision support were also applied frequently.

Telemedicine DHTs (n=10) include those that enable consultation for case management and knowledge exchange between health care providers or those, such as the Vula mobile app, (Supplement 2) that enable diabetes and mental health–related consultations between remote health care providers and doctors during real-time visits with patients. DHTs, including text messages, phone calls, and mobile apps, were also used to monitor and inform patients about adherence to treatment and health management in relation to diabetes, cancer, and general NCDs.

Less frequent was the application of DHTs enabling laboratory and diagnostics imaging management (n=6). The ones included involved establishment and expansion of telepathology services that allow efficient and secure transmission of clinical pathology images between providers at a large hospital and between health centers participating in the respective access programs, targeting primary childhood and lung cancer, as well as cardiovascular disease and diabetes. Information about the additional DHTs that were applied less frequently (i.e., health provider communication [n=5], referral coordination [n=4], and prescription and medication management [n=1]) can be found in Supplement 2.

### DHTs Targeting Data Services

DHTs targeting data services mostly focused on enabling data collection, management, and use (n=22). The technologies include an online platform synthesizing data of all practicing and registered oncology professionals from East, Central, and Southern Africa (e.g., AMPATH). Moreover, digital data collection tools help securely register and track population and patient data. One mobile health care platform, M-TIBA, developed by PharmAccess Foundation, Safaricom, and CarePay and used by Ngao Ya Afya in Kenya, functions as a mobile health wallet that also generates data on costs and outcomes of care at an individual patient level. Another digital software used by the Save Her Ghana project collects data for inventory management.

Six programs included DHTs that enable data exchange across systems that ensure secure communication and data governance, such as within hospital departments; between hospitals, collaborating partners, and lower-level centers; and between patients, providers, and payers. Digital software to upgrade and implement CanReg 5, a multiuser platform and open-source tool to input, store, check, and analyze cancer registry data, was also an integral part of the Secure the Future access program in Swaziland. Lastly, DHTs enabling location mapping were used by the Remember I Love You project for dementia patients in China and by AMPATH for oncologists in East, Central, and Southern Africa.

### DHTs Targeting Clients

Client-focused DHTs often entail targeted client communication (n=15). Technologies include digital patient education materials on disease and treatment, disease-specific websites and social media channels, disease awareness SMS campaigns, phone-based treatment reminders, disease management support and free-of-charge medicine arrangements, artificial intelligence–enabled chatbots for patients, and electronic toolkits in multiple languages and cultural adaptations developed for Android and iOS. The Mobile Healthcare Field Clinic Services included a DHT aimed at more untargeted client communication, namely general awareness campaigns on immunization and disease prevention in selected communities in Tanzania.

Client-focused DHTs often entail targeted client communication that deliver patient education information, treatment reminders, and disease management support.

Digital health technologies targeting clients also include the mobile app for personal health tracking (n=1) developed under the Sparta project that enables patients receiving therapies to track and self-monitor diet, weight, exercise, and test results. Novartis Access and Ngao Ya Afya included DHTs that enable clients to look up their own health information (n=2).

Lastly, the mobile wallet M-TIBA facilitates financial transactions for clients related to health service delivery. The wallet allows people to save money and receive insurance or other entitlements ring-fenced for health care that can be spent in connected clinics. By using M-TIBA, the Ngao Ya Afya program gives patients access to discounted diabetes and hypertension consultations, medical tests, and discounted company medicines.

### DHTs Targeting Health System Management

Programs using health system management DHTs (n=9) were largely focused on strengthening supply chain management (n=3), which included technologies for management of oncology and general NCD medicine stock and support functions for demand planning and inventory management. The DHT used in Blueprint for Innovative Healthcare Access also enables rapid communication and solutions between the different hierarchical levels of the supply chain, such as collaboration between facilities.

Other DHTs targeted human resource management (n=2). The Integrated Cancer Curriculum program in Kenya included a DHT aimed at training primary health care providers in cancer care management. Once trained, each health provider is certified and becomes a resource for the National Cancer Institute and Ministry of Health Kenya. The DHT then allows for identification of trained providers for cancer management training opportunities with other primary health care providers. The AMPATH program also used an online platform with an accessible database of all practicing and registered oncology professionals, including their qualifications and contact information, within East, Central, and Southern Africa.

Two programs applied DHTs for health financing (n=2), including Rightmax software for management of funds used by the Secure the Future Uthukela lung cancer program in South Africa and the mobile wallet M-TIBA, used by Ngao Ya Afya to allow diabetes and hypertension patients to manage health budgets and expenditures.

A single program applied a DHT that enables digital birth registration and strengthening of civil registration and vital statistics in Kenya. Another program, Instrumental Access, developed by the U.S.-based nonprofit organization Seeding Labs and implemented across LMICs in multiple regions, included an online portal for local universities to submit requests for equipment to support their local general NCD research programs.

## DISCUSSION

There is a need for more research and information on the use of DHTs within health systems, particularly in LMICs.^7^ To our knowledge, this article is the first to provide a comprehensive review of pharmaceutical industry–led access programs’ use of DHTs. This study contributes to expanding the evidence base on how DHTs can be applied to improve access to NCD care in LMICs.^11^^,^^16^^–^^18^

Overall, the programs that applied DHTs were similar to programs that did not use DHTs in terms of country of implementation and targeted disease areas. However, industry-led access programs targeting type 2 diabetes in LMICs were significantly more likely to apply DHTs than not. This finding may be explained by the fact that DHTs to support people with type 2 diabetes have been growing rapidly and are among the most commonly available.^19^ Yet, pharmaceutical companies’ widespread decision to apply DHTs to target type 2 diabetes also emphasizes to national stakeholders the availability and potential opportunities of DHTs to promote access to type 2 diabetes care in LMICs. Type 2 diabetes is a disease that requires a high level of both patient and provider monitoring of nutrition, exercise, weight control, blood glucose, and insulin levels.

In terms of access strategy, programs targeting health service delivery and community awareness were surprisingly less likely to apply DHTs. Digital technologies evidently exist to facilitate community awareness, such as providing communities and patients with health-related information on NCD prevention, as well as health service delivery, such as screening, diagnosis, and treatment. Our finding may reveal an opportunity for sharing best practices among stakeholders on how DHTs can be used effectively to facilitate community awareness or health service delivery. For example, only 2 companies used DHTs to provide screening for cancer and mental health disorders, respectively. For programs focusing on patient medicine donation and pricing schemes, DHTs may be less relevant.

We used a combination of the Access Observatory Strategy Taxonomy and the WHO Digital Health Classification Taxonomy to present the employed DHTs by health system function (i.e., health service delivery, health service strengthening, community awareness) and target (i.e., client, provider, data services, or health system managers). Among the identified DHTs, most were applied in relation to health service strengthening followed by community awareness and health service delivery. The programs focused on health service strengthening, health service delivery, and community awareness mostly used DHTs to target health care providers. This finding aligns with existing literature that attests to the potential of DHTs to achieve health workforce improvements in LMICs.^20^ The ongoing transition in disease epidemiology from infectious diseases to NCD care in LMICs compounded by health worker shortages and escalating costs of delivering health services calls for innovative ways to enhance capacity among health providers. The reviewed DHTs reveal existing opportunities that potentially can be scaled to upskill health providers, enabling them to facilitate patient management and treatment and to provide accurate referral and follow-up which is critical for NCD management.

The reviewed DHTs reveal existing opportunities that can be scaled to upskill health providers, enabling them to facilitate patient management and treatment and to provide accurate referral and follow-up.

Data-focused DHTs were employed in more than half of the programs that used DHTs. These DHTs hold tremendous power to generate real-world data on the delivery of care, clinical outcomes, and epidemiology of NCDs, which can be harnessed to help address the disease burden of NCDs at global, national, regional, and local levels.^21^ Moreover, companies implementing these DHTs may benefit from the generated intelligence to further develop their business in emerging markets, e.g., by identifying influential risk factors, unmet needs, and high-risk populations. The large amounts of data being collected from DHTs applied in pharmaceutical industry–led programs give pharmaceutical companies power over information, and public payers such as governments and citizens should consider to what extent this data may be used for commercial activities.^22^ In its *Global Strategy on Digital Health*, the WHO stresses the need for a strong legal and regulatory base to protect the integrity and availability of data.^7^ It recommends sharing of collected data to support the planning, commissioning, and transformation of health care services.^23^ By ensuring secure ways of data sharing and transparency between pharmaceutical companies, governments, academic centers, and other stakeholders, real-world evidence collected through DHTs can yield greater impact in preventing and managing NCDs.^21^

For client-focused DHTs, we found that pharmaceutical companies largely focus on delivering disease-specific communication and general awareness to patient populations. This trend is likely connected to the rapid increase in mobile phones, smartphones, Internet, and social media across LMICs, which provides an opportunity to digitally educate patients suffering from NCDs in an effective, efficient, and cost-saving way. Digital medical reminders, prescription assistance, advice, and support to change lifestyle behaviors and manage disease and pharmaceuticals have proven to be effective in terms of improving NCD risk factors and health outcomes,^24^ and they contribute to health equity by enabling patients who live in rural areas to receive the same information as those in more accessible areas.

Lastly, only a few DHTs targeted health system management (i.e., supply chain, human resource management, and health financing). Management of these aspects within health systems is critical, particularly in low-resource settings where efficient use of resources is necessary to achieve health-related goals. Strengthening core health system functions, such as management, are likely to be less of a priority for pharmaceutical companies compared with, for example, enhancing provider knowledge and patient awareness, as these may yield more rapid results in terms of market expansion. Nonetheless, most data-focused DHTs, particularly those involving data collection, use, and management, also facilitate health system management in LMICs. By introducing digital data collection tools, such as DHIS2,^9^^,^^25^ administrative matters are simplified, which in turn helps health managers foresee local disease burden and plan for health services demand, stocks, and disease outbreaks.

### Future Digital Health Implementation Considerations

This article found that a plethora of technologies is being implemented within and across LMICs. The general descriptions of the DHTs in program reports made it difficult for the authors to assess whether similar technologies described in multiple program reports were identical. Specifically, many DHTs implemented by various companies used similar-sounding digital platforms for provider training, tools for collecting patient records, and data systems. Efforts have been made to strengthen the coordination of ICT investments made by international NGOs to reduce duplicative efforts^26^; however, it is unclear whether similar efforts have been made for industry-led DHT implementations. Previous studies indicate that digital health solutions are usually developed in isolation, and undertaken in stand-alone, vertical projects that provide limited evidence on their impact on health systems.^9^^,^^26^ The lack of coordination leads to a fragmented digital health landscape, with multiple pilot interventions without sustainability plans.^12^^,^^17^^,^^25^ Poor integration and co-existence of different systems with similar objectives may negatively affect local health systems by overburdening health providers and detracting resources from local health priorities and needs.^9^^,^^26^ Transparent and detailed reporting on DHT implementation may facilitate partnerships between companies, governments in LMICs, and other organizations implementing DHTs that can decrease duplicative efforts and promote more effective and sustainable solutions to be integrated into local health systems.

Moreover, few program reports in the Access Observatory repository include data values demonstrating DHT implementation outcomes and impact in terms of improving NCD care. Most of the program reports in the Access Observatory provide data on output indicators (i.e., number of people trained, population exposed to communication activities, population screened). Meanwhile, there is limited data on outcomes and impact indicators (i.e., patients with cancer remission, mortality rates, patient satisfaction).^27^ Information on costs involved with the development and implementation of DHTs are also not available in the program reports. Further exploration of costs and the impact of DHTs on NCD care in LMICs is needed to assess whether they are effective, feasible, and sustainable in this context. This is critical to ensure responsible allocation of resources “to promote health and reduce inequalities … and to promote the appropriate integration and use of technologies,” as stated in WHO’s recommendations on digital interventions for health system strengthening.^23^^,^^26^^,^^28^ To facilitate improvements in digital health governance, policy makers and investors should make the case for robust measurement and transparent reporting on industry-led DHT implementations in LMICs.

To facilitate improvements in digital health governance, policy makers and investors should make the case for robust measurement and transparent reporting on industry-led DHT implementations in LMICs.

### Limitations

The following limitations need to be taken into consideration. The focus on NCDs in the Access Observatory database may likely have caused companies to report solely on DHTs that target NCDs specifically. Other DHTs that may be in use by the industry to address broader health system challenges may thus not have been captured, despite these potentially having indirect yet significant effects on NCDs in LMICs. Moreover, shortcomings exist in company reporting on their access programs, including their descriptions of applied DHTs. Limited information is provided on functionalities of the DHT, potential barriers and facilitators of DHT implementation, developers, costs, fruitful partnership models, and level of integration in local health systems. The program reports were not intended to collect information on DHTs specifically; thus, more comprehensive insights into the mentioned aspects of DHTs use are encouraged in future research, ideally through in-depth case studies of pharmaceutical companies’ application of DHTs. The reports’ shortcomings may have affected the authors’ ability to correctly identify whether DHTs were applied in the access program. Through the systematic review of 101 access program reports, we found that less than half of the reviewed programs employed DHTs. Naturally, not all access programs are prone to apply DHTs. Secondly, the limited level of detail provided in the reports may have hindered the authors’ ability to classify the DHTs accurately in terms of the WHO Digital Health Classification Framework. Thirdly, the test of statistically significant differences between programs using DHTs versus those not using DHTs may have been influenced by the small sample; an effect that failed to be significant (*P*<.05) could prove significant in a larger sample. Finally, the reporting of access programs to the Access Observatory is voluntary; as a result, the sample of access programs studied is not representative of all pharmaceutical industry–led access initiatives. However, the Access Observatory contains reports from 19 of the world’s leading pharmaceutical companies, differing in size, product portfolios, and geographical reach, and thus serves as the largest and most comprehensive repository of pharmaceutical industry–led access programs with data on DHT use. Currently, it is the best available data source to study these programs.

## CONCLUSION

The pharmaceutical industry plays a significant role in achieving the United Nations Sustainable Development Goals, including those related to NCDs, and thus, their efforts are important to explore. The DHTs implemented by pharmaceutical companies addressing access barriers should be recognized and so should their efforts in reporting on these and making the information publicly available in the Access Observatory. However, in the future, companies should aim and be held accountable to provide more robust measurement and reporting on their digital health implementation efforts. Transparency on the use and effect of DHTs can reduce the number of duplicative and redundant efforts and facilitate partnerships that can have greater impact on reducing NCDs in LMICs.

## Supplementary Material

GHSP-D-22-00072-supplement.pdf

## Notes

**Peer Reviewed**

**First published online:** September 26, 2022.
