# Supplementary material for: Digital Health Technologies Applied by the Pharmaceutical Industry to Improve Access to Noncommunicable Disease Care in Low- and Middle-Income Countries
Source: Glob Health Sci Pract. 2022 Oct 31;10(5):e2200072. doi: 10.9745/GHSP-D-22-00072 (PMC9622287; doi:10.9745/GHSP-D-22-00072)
Supplement: GHSP-D-22-00072-supplement.pdf [file GHSP-D-22-00072-supplement.pdf]

Supplement to: Heerdegen ACS, Cellini CM, Wirtz VJ, Rockers PC. Digital health technologies applied by the pharmaceutical industry to improve access to non-communicable disease care in low-and middle-income countries. *Glob Health Sci Pract*. 2022;10(5):e2200072. https://doi.org/10.9745/GHSP-D-22-00072

Supplement 1. The WHO Digital Health Classification Framework

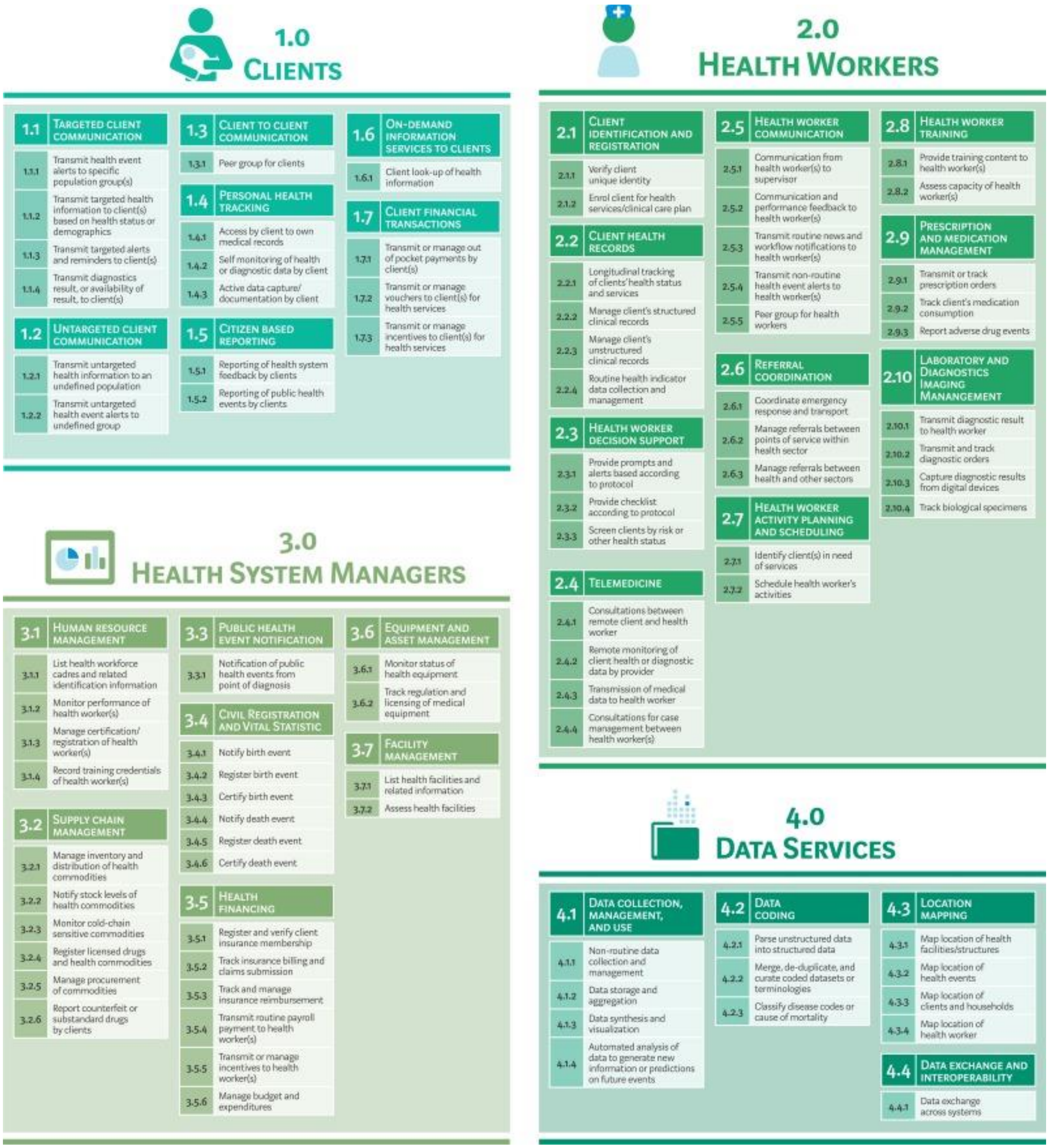

Supplement to: Heerdegen ACS, Cellini CM, Wirtz VJ, Rockers PC. Digital health technologies applied by the pharmaceutical industry to improve access to non-communicable disease care in low- and middle-income countries. *Glob Health Sci Pract*. 2022;10(5):e2200072. https://doi.org/10.9745/GHSP-D-22-00072

Supplement 2. Overview of each of the DHTs, relevant strategies, and WHO classifications

	Company name	Program & Disease Area	Country	Digital Health Technology	WHO Classification	Program Strategy
**Sub-Saharan Africa**						
1	Roche	Save Her, Ghana	Ghana	• Digital software for inventory management and program monitoring aiming to improve efficiency in the supply chain, track the patient journey, and provide insights and evidence to the government for policy consideration.	**2.0:** 2.2<br>**3.0**: 3.2<br>**4.0:** 4.1	Supply Chain
2	BMS	Secure the Future - Lung Cancer in Kenya	Kenya	• Electronic risk-factor screening tools for lung cancer.<br>• Telepathology with the participating centers for pathology reviews	**2.0:** 2.3, 2.10	Health Service Strengthening
3	Takeda	Blueprint for Innovative Healthcare Access	Kenya	• Expansion of Telemedicine and Telepathology services at the Meru Hospital<br>• Point of Care electronic patient record systems for secure and accurate recording of patient data to provide accurate referrals and follow-ups.<br>• LEAP mHealth Platform delivering eLearning content on NCD management and patient support to community health workers.<br>• Mobile Jamii Afya Link (M-Jali) increases and improves household/community level data on NCDs through community health workers.<br>• Technology for managing stock which enables visibility of available medicine at facility level, which will support demand planning and improve inventory management. The DHT will disseminate real-time information to key stakeholders—enabling rapid response to and resolution of identified challenges and improving communication between the different hierarchical levels of the supply chain—including collaboration between facilities to address supply challenges.	**2.0:** 2.2, 2.4, 2.8, 2.10<br>**3.0:** 3.2<br>**4.0:** 4.1	Community awareness, Health service strengthening, Supply Chain

Supplement to: Heerdegen ACS, Cellini CM, Wirtz VJ, Rockers PC. Digital health technologies applied by the pharmaceutical industry to improve access to non-communicable disease care in low- and middle-income countries. *Glob Health Sci Pract*. 2022;10(5):e2200072. https://doi.org/10.9745/GHSP-D-22-00072

	Company name	Program & Disease Area	Country	Digital Health Technology	WHO Classification	Program Strategy
4	BMS	Celgene AMPATH Oncology Partnership	Kenya	• Electronic medical records with decision support, prescription assistance, and pharmaceutical management to enable timely ordering of medications and inventory control.<br>• Data sharing and patient education website for people suffering from Multiple Myeloma in Kenya.<br>• Adverse drug reaction monitoring through phone call follow-ups and timely management offered in consultation with hemato-oncologists.	**1.0:** 1.1, 1.4<br>**2.0:** 2.9, 2.3, 2.2<br>**3.0:** 3.2<br>**4.0:** 4.1	Community Awareness; Health Systems Strengthening; Health Service Delivery; Supply Chain
5	Takeda	Cancer Alliance for Sub-Saharan Africa	Kenya	• Patient data collection and management system to improve tracking of cancer incidence and type, and inform policy-making	**2.0:** 2.2<br>**4.0:** 4.1	Health service strengthening
6	Takeda	Chronic Care Program in Sub-Saharan Africa	Kenya	• Virtual training of health workers in rural areas through the LEAP mHealth platform. LEAP delivers training through SMS and audio files using basic phone technology, as well as rich content such as animations and illustrations to smartphones.	**2.0:** 2.2, 2.6, 2.8	Health service strengthening
7	Takeda	Digital Birth Registration in Kenya	Kenya	• Registration of births using a digital model, developed by Swedish NGO Shifo Foundation	**3.0:** 3.4<br>**4.0:** 4.1	Community awareness
8	Sanofi	Ngao Ya Afya	Kenya	• M-TIBA is a mobile health care platform that connects patients, payers, and providers through a mobile health wallet, which can be used by anyone with access to a simple mobile phone. The wallet allows people to save money and receive insurance or other entitlements, ring-fenced for healthcare that can be spent in connected clinics. M-TIBA enables direct and targeted reach of large groups of patients with entitlements at virtually no transaction costs. The platform generates data along the care process that creates transparency on costs and outcomes of care, at an individual patient level. Data can be used to provide smart, real-time feedback to patients, providers, and payers and improve the value of care.	**1.0:** 1.6<br>**3.0:** 3.5<br>**4.0:** 4.1, 4.4	Health service strengthening
9	Takeda	Integrated Cancer Curriculum	Kenya	• Mobile and online training courses and learning, including mentorship and knowledge sharing for primary care physicians and community health workers. Community health workers can get learning delivered through SMS and voice messaging. Health workers are certified following participation in the training, which grants recognition and legitimacy with	**2.0:** 2.8<br>**3.0:** 3.1	Community Awareness, Health service strengthening

Supplement to: Heerdegen ACS, Cellini CM, Wirtz VJ, Rockers PC. Digital health technologies applied by the pharmaceutical industry to improve access to non-communicable disease care in low- and middle-income countries. *Glob Health Sci Pract*. 2022;10(5):e2200072. https://doi.org/10.9745/GHSP-D-22-00072

	Company name	Program & Disease Area	Country	Digital Health Technology	WHO Classification	Program Strategy
				institutions across Kenya. Once trained, trainers will be identified and become a resource to the National Cancer Institute and Ministry of Health Kenya to support cancer management training of other primary healthcare providers.		
10	Takeda	Patient Support and Palliative Care Training in SSA	Kenya	• 26 digital learning modules of the Integrated National Curriculum on patient support and palliative care to train primary healthcare professionals and community health workers. Training is facilitated through the m-health LEAP platform, which is a scalable and integrated interactive learning solution that offers continuous training opportunities, peer collaboration, and real-time evaluation reports.<br>• The program also makes use of Mobile Jamii Afya Link (m-JALi) to enable community health workers to accurately and efficiently collect and manage patient data at the household, community, and facility levels.	**2.0:** 2.2, 2.8<br>**4.0:** 4.1	Health service strengthening
11	BMS	Secure the Future - Gauteng Province Africa Lung Cancer	South Africa	• Electronic database for patient records to initiate research on the burden of lung cancer at three different hospitals	**2.0:** 2.2<br>**4.0:** 4.1	Health Service Strengthening
12	BMS	Secure the Future - Kimberly Hospital Complex, South Africa	South Africa	• Data system, data collection, and utilization capabilities within Kimberly Hospital Complex HC and collaborating partners to improve patient quality of care, monitor service delivery, and report hospital cancer data.	**4.0:** 4.1, 4.4	Health Service Strengthening
13	BMS	Secure the Future - Kwazulu Natal, South Africa Lung Cancer	South Africa	• Electronic risk-factor screening tools for lung cancer.<br>• Establish telepathology with the participating centers for pathology reviews.	**2.0:** 2.3, 2.10	Health Service Strengthening
14	BMS	Secure the Future - Uthukela, KZN, South Africa Cancer	South Africa	• Facebook, Twitter, and Instagram to create community awareness about common cancers, HIV, TB, and other NCDs in people aging with HIV.<br>• Tablets for data collection and patient follow-up.<br>• Use of NHLS website to access results.<br>• Software Rightmax system for management of funds	**3.0:** 3.5<br>**4.0:** 4.1	1) Community awareness 2) HSS
15	BMS	Project ECHO for Cancer Care	South Africa	• Telemedicine system ("teleECHO clinics") for knowledge-sharing network, led by expert teams who use multi-point videoconferencing and telementoring to conduct "virtual	**2.0:** 2.4, 2.5, 2.8	Health Service Strengthening

	**Company name**	**Program & Disease Area**	**Country**	**Digital Health Technology**	**WHO Classification**	**Program Strategy**
				clinics" with community providers to enable community-based specialists, primary care doctors, nurses, and other clinicians to learn to provide excellent specialty care to cancer patients in their communities. The technology used for this includes videoconferencing hardware & software, video recording system, and a YouTube-like website/archive. The program also introduces "iEcho" & "ECHO Health" systems for clinic management.		
16	Eli Lilly	Tshwane Insulin Project	South Africa	• Vula Mobile app to facilitate communication between health care professionals. With remote technical assistance and mentorship, primary care doctors, and professional nurses at local clinics will be able to benefit from the knowledge and expertise of diabetes specialists.	**2.0:** 2.5, 2.8	Health service strengthening
17	BMS	Secure the Future - Lung Cancer in Swaziland	Swaziland<br><br>Lung Cancer	• Upgrade and implement the CanReg 5 software. CanReg5 is an open-source tool to input, store, check and analyze cancer registry data.	**2.0:** 2.8, 4.1, 4.4	Community awareness
18	Sanofi	Sanofi Mental Health Program FAST - South Africa	South Africa	• Virtual interactive workshops, or face-to-face workshops, with e-learning to upskill 500 primary HCPs across all 9 provinces<br>• Trained HCPs linked via the Vula mobile app to public sector specialists at hospitals to allow mHealth consultations and up-referrals.	**2.0:** 2.4, 2.6, 2.8<br>**4.0:** 4.4	Health Service Strengthening
19	BMS	Secure the Future - Tanzania	Tanzania<br><br>Lung Cancer	• Electronic referral tool from lower-level centers to Medical center.<br>• Electronic risk-factor screening tools for lung cancer.<br>• Establish telepathology with the participating centers for pathology reviews.<br>• Establish/strengthen cancer registries to include lung cancer and collect all epidemiology data.	**2.0:** 2.6, 2.10<br>**4.0:** 4.1, 4.4	1) Community awareness 2) Health Service Strengthening
20	Daichii Sankyo	Mobile Healthcare Field Clinic Services	Tanzania	• Information systems and tools for communication campaigns to spread awareness about immunization and disease prevention in communities.	**1.0:** 1.2<br>**2.0:** 2.8	Community Awareness
21	Merck KGaA	Merck Community Awareness Program	Angola, Ethiopia, Ghana, India, Indonesia, Kenya,	• Social media campaign for prevention and early detection of cancer and diabetes, including videos and posters. Cancer awareness videos on youtube in English and local languages.	**1.0:** 1.1<br>**2.0:** 2.3, 2.5, 2.8	Community awareness

Supplement to: Heerdegen ACS, Cellini CM, Wirtz VJ, Rockers PC. Digital health technologies applied by the pharmaceutical industry to improve access to non-communicable disease care in low- and middle-income countries. *Glob Health Sci Pract*. 2022;10(5):e2200072. https://doi.org/10.9745/GHSP-D-22-00072

	Company name	Program & Disease Area	Country	Digital Health Technology	WHO Classification	Program Strategy
			Mozambique, South Africa, Tanzania, Uganda			
22	Takeda	AMPATH Oncology Preceptorships and Telemedicine	Kenya, Rwanda, Uganda, Tanzania	• Video-call to facilitate one-on-one consultations with doctors in healthcare facilities outside of urban areas, enabling patients to receive immediate and high-quality access and care. Telemedicine supports the extension of training courses, so healthcare providers can consult with doctors during real-time visits with patients. The platforms also increase collaborative trainings within the East, Central, and Southern Africa communities of oncology professionals.<br>• An online platform with an accessible database of all practicing and registered oncology professionals from East, Central, and Southern Africa, which will include classification of services by location, qualifications of oncologists, and healthcare professionals' relevant contact information. The platform shares standardized curriculum and protocols for cancer treatments, as well as provides patient information on cancer management, which will include nutrition, management of side effects of cancer, and locally available patient support resources.	**1.0:** 1.1<br>**2.0:** 2.3, 2.4, 2.5, 2.8<br>**3.0:** 3.1<br>**4.0:** 4.1	Health Service Strengthening
23	Novo Nordisk	Base of the Pyramid	Ghana, Nigeria, Kenya, Morocco, Senegal	• Supporting clinics with diabetes-specific data tools to register and hold patient data	**2.0:** 2.2<br>**4.0:** 4.1	Health Service Strengthening
24	Sanofi	FAST – eLearning<br>.	Mali, Senegal	• eLearning platform, including 12 online modules which healthcare provider learners can complete at their own pace, with interactive webinars organized by local psychiatrists.	**2.0:** 2.8	Health Service Strengthening
	**East Asia & Pacific**					
25	Daichii Sankyo	Cultivating Healthcare Workers in China	China	• Information system and tools (e.g. a copy of Integrated Management of Childhood Illness guidelines) for healthcare workers.	**2.0:** 2.8	Health service strengthening

	Company name	Program & Disease Area	Country	Digital Health Technology	WHO Classification	Program Strategy
26	Eisai	Remember I Love You	China	• The "Yellow Wristband" social media platform targets the general population including the elderly. The platform includes articles on dementia self-assessment, drug compliance, and a memory clinic list are published regularly and patients can apply for yellow wristband, a wristband with GPS function to track dementia patients. The yellow wristband also contains information on dementia patients which could be used to find their home and/or be in touch with their contacts if lost.	**1.0:** 1.1<br>**4.0:** 4.3	Community Awareness
	**South Asia**					
27	Merck & Co	SPARSH Healthline	India	• Phone-based complementary diabetes management patient support program for patients on Merck Medicine to help improve patient's diabetes control and health outcomes via counseling them on their disease and its management, self-management of diet, exercise, and other lifestyle changes, and adherence and compliance to prescribed treatment plan.	**1.0:** 1.1	Health Service Strengthening
28	Roche	The Blue Tree, India	India	• Telephone calls by program coordinator to remind patients to adhere to treatment (i.e., reminder calls 48 hours before infusion and 48 hours post-infusion to confirm completion).	**1.0:** 1.1<br>**2.0:** 2.4	Community awareness, Health Service Delivery, Financing, Price Scheme
29	Pfizer	SMARThealth Extend	India<br>Indonesia	• Mobile device-based clinical decision support system (CDSS) for use by healthcare providers; and integration of this system within the existing public primary healthcare sector.	**2.0:** 2.3<br>**4.0:** 4.1, 4.4	1) Community awareness 2) Health Service Strengthening, 3) Health Service Delivery
30	Roche	UNMOL: Access to Cancer Medicines, Pakistan	Pakistan	• Data management system providing information regarding treatment cycles to improve the patient journey. After signing the patients to the program, Dimension Research makes telephone calls to all enrolled patients to arrange free-of-charge medicine. Similarly, they call the patients to verify if the patient has started the treatment and then record that information in the system.	**1.0:** 1.1<br>**2.0:** 2.2, 2.4<br>**4.0:** 4.1	Health Service Strengthening
31	Pfizer	Abundant Health	Vietnam	• CommCare, a data collection and case management tool, facilitates patient management and treatment of diabetes and hypertension, as well as supports quality data management.	**2.0:** 2.2<br>**3.0:** 3.6<br>**4.0:** 4.1	Health Service Strengthening

Supplement to: Heerdegen ACS, Cellini CM, Wirtz VJ, Rockers PC. Digital health technologies applied by the pharmaceutical industry to improve access to non-communicable disease care in low- and middle-income countries. *Glob Health Sci Pract*. 2022;10(5):e2200072. https://doi.org/10.9745/GHSP-D-22-00072

	Company name	Program & Disease Area	Country	Digital Health Technology	WHO Classification	Program Strategy
			Hypertension and Diabetes			
32	Sanofi	Sanofi Mental Health Program FAST - Myanmar	Myanmar<br><br>Mental Health	• Providing GPs with electronic tablets, equipped with an interactive version of the World Health Organization Mental Health Gap Action Program intervention guide, and e-medical records, to manage patients and potentially seek advice from psychiatrists for difficult cases.<br>• Providing CHWs with smartphones equipped with interactive screening questionnaires and empowering them in their pivotal role in informing the population, combating the misbeliefs surrounding mental disorders, and identifying people with these diseases so that they can be referred to GPs.	**2.0:** 2.2, 2.3, 2.4, 2.6<br>**4.0:** 4.1	Health Service Strengthening, Health Service Delivery
	**Latin America and the Caribbean**					
33	Roche	Pink Consulting Rooms, Colombia	Colombia<br><br>Breast Cancer	• Data information tool to decrease time and process along the patient journey, namely between suspected diagnosis, confirmed diagnosis, and treatment for breast cancer patients in Columbia. The system encourages monitoring and patient support throughout the entire journey.	**2.0:** 2.2<br>**4.0:** 4.1	Health Service Strengthening
34	Sanofi	My Child Matters – Paraguay	Paraguay	• Phone calls to follow up on patients who do not attend appointments<br>• Development of an electronic classification tool to assess "risk of abandonment" and identify the families with social risk that require more support to carry out the treatment.<br>• Community awareness campaigns via computers, internet, media, radio, TV, and newspapers<br>• eLearning platform for health provider training in early diagnosis of childhood cancer.<br>• Development of a data registration system to track patient data.	**1.0:** 1.1<br>**2.0:** 2.3, 2.8<br>**4.0:** 4.1	Health service strengthening, health service delivery
	**Implemented across multiple regions**					
35	Sanofi	Kids and Diabetes in School	Argentina, Brazil, Egypt, Hungary, India, Japan, Pakistan, Phillippines,	• Electronic toolkit, developed for Android and an IOS version for iPad, providing awareness and information on the management of type 1 diabetes in children and the prevention of type 2 diabetes for teachers, school staff including school nurses, parents (including parents of a child with diabetes) and children (6-14 years old). It is available in multiple languages	**1.0:** 1.1<br>**2.0:** 2.8	Community Awareness

	Company name	Program & Disease Area	Country	Digital Health Technology	WHO Classification	Program Strategy
			Poland, United Arab Emirates	and cultural adaptations, free of charge on the IDF KiDS website (https://kids.idf.org/).		
36	Sanofi	My Child Matters - Telepathology for Childhood Cancer Diagnosis	Benin, Burkina Faso, Cameroon, Cote d'Ivoire, the Democratic Republic of the Congo, Mali, Niger, Senegal	• ipath: Telepathology offering efficient communication between physicians. It consists of the transmission of digital images from a microscope combined with a connected camera to a shared online platform.	**2.0:** 2.5, 2.10	Health service strengthening
37	Takeda	Instrumental Access Program: Building Research Capacity in LMICs	Benin, Cameroon, Dominican Republic, India, Liberia, Malawi, Namibia, Nigeria, Peru, South Africa, Tanzania, Ukraine, Vietnam, Zambia, Zimbabwe	• Seeding Labs online portal for local universities to submit requests for equipment to support their local research programs.	**3.0:** 3.6	Product Development Research
38	Takeda	R&D Access to Medicines Employee Fellowship Program	Kenya, Rwanda, Tanzania, Haiti	• Virtual knowledge sharing, mentoring, and training of local stakeholders including NGOs, academia, local hospitals, and healthcare workers to support and build healthcare capacity in areas such as clinical care, epidemiology, training, R&D project management, and supply chain.	**2.0:** 2.8	Health service strengthening

Supplement to: Heerdegen ACS, Cellini CM, Wirtz VJ, Rockers PC. Digital health technologies applied by the pharmaceutical industry to improve access to non-communicable disease care in low- and middle-income countries. *Glob Health Sci Pract*. 2022;10(5):e2200072. https://doi.org/10.9745/GHSP-D-22-00072

	Company name	Program & Disease Area	Country	Digital Health Technology	WHO Classification	Program Strategy
39	Merck & Co	Sparta	Australia, Austria, Belgium, Brazil, Germany, Greece, India, Indonesia, Ireland, Malaysia, Mexico, Oman, Philippines, Russia, Singapore, Sweden, Switzerland, Taiwan, Thailand, United Arab Emirates, Vietnam	• A set of health technology platforms (website, nurse call center, SMS and email campaigns on health tips, mobile app for tracking diet, exercise, test results, weight, goals) to support patients on Merck Sharp & Dohme (MSD) therapy. Patients receive education on their therapy and disease as well as guidance on diet, exercise, and lifestyle modifications that can help them achieve better health outcomes.	**1.0:** 1.1, 1.4<br>**2.0:** 2.2, 2.4<br>**4.0:** 4.1	1) Community Awareness, 2) Health Service Strengthening, 3) Health Service Delivery
40	Merck KGaA	Merck Capacity Advancement Program	Angola, Bangladesh, Cambodia, Cameroon, Central African Republic, Congo, Cote d'Ivoire, Equatorial Guinea, Ethiopia, Ghana, India, Indonesia,	• Diabetes awareness SMS campaign	**1.0:** 1.1	Community awareness

	Company name	Program & Disease Area	Country	Digital Health Technology	WHO Classification	Program Strategy
			Kenya, Liberia, Malawi, Mali, Mozambique, Myanmar, Nepal, Nigeria, Rwanda, Senegal, Sierra Leone, South Africa, Sri Lanka, Tanzania, Uganda, United Arab Emirates, Zambia, Zimbabwe			
41	Merck KGaA	Merck Cancer Access Program	Botswana, Egypt, Ethiopia, Ghana, India, Kenya, Liberia, Namibia, Sierra Leone, South Africa, Tanzania, Uganda, Zambia	• Social media community prevention & early diagnosis awareness campaigns to share accurate cancer information to help in tackling myths, misconceptions, and stigma.	**1.0:**1.1	1) Community awareness
42	Novartis	Novartis Access	Kenya, Uganda, Nigeria, Pakistan, El Salvador,	• Digital health technologies enabling community members to receive education on NCDs, information about their own health and management plans, and additional information to empower their health-seeking behavior.	**1.0:** 1.1 **2.0:** 2.3, 2.4, 2.10	1) Community awareness, 2) Health Service Strengthening

Supplement to: Heerdegen ACS, Cellini CM, Wirtz VJ, Rockers PC. Digital health technologies applied by the pharmaceutical industry to improve access to non-communicable disease care in low- and middle-income countries. *Glob Health Sci Pract*. 2022;10(5):e2200072. https://doi.org/10.9745/GHSP-D-22-00072

	Company name	Program & Disease Area	Country	Digital Health Technology	WHO Classification	Program Strategy
			Cameroon, Ethiopia			
43	Novo Nordisk	Changing Diabetes in Children	Bangladesh, Cambodia, Cameroon, Cote d'Ivoire, the Democratic Republic of the Congo, Ethiopia, Guinea, India, Kenya, Myanmar, Senegal, Sudan, Tanzania, Uganda	• Social media campaigns on diabetes awareness through local diabetes associations.<br>• Patient registry system to facilitate systematic data collection and patient follow up	**1.0:** 1.1<br>**2.0:** 2.2<br>**4.0:** 4.1	1) Community awareness, 2) Health Service Strengthening
